# The Efficacy and Safety of Single-Strain Probiotic Formulations Containing *Bifidobacterium lactis* or *Bacillus coagulans* in Adult Patients with Irritable Bowel Syndrome—A Randomized Double-Blind Placebo-Controlled Three-Arm Interventional Trial

**DOI:** 10.3390/jcm12144838

**Published:** 2023-07-22

**Authors:** Barbara Skrzydło-Radomańska, Beata Prozorow-Król, Anetta Kurzeja-Mirosław, Halina Cichoż-Lach, Katarzyna Laskowska, Emilia Majsiak, Joanna B. Bierła, Sowińska Agnieszka, Bożena Cukrowska

**Affiliations:** 1 Department of Gastroenterology, Medical University of Lublin, Jaczewskiego 8, 20-950 Lublin, Poland; barbara.radomanska@gmail.com (B.S.-R.); prozorow1@wp.pl (B.P.-K.); anettakm@op.pl (A.K.-M.); lach.halina@wp.pl (H.C.-L.); katarzynalaskowska@go2.pl (K.L.); 2 Department of Health Promotion, Faculty Health of Sciences, Medical University of Lublin, Staszica 4/6, 20-081 Lublin, Poland; emiliamajsiak@umlub.pl; 3 Department of Pathomorphology, The Children’s Memorial Health Institute, Aleja Dzieci Polskich 20, 04-730 Warsaw, Poland; j.bierla@ipczd.pl (J.B.B.); a.sowinska@ipczd.pl (S.A.)

**Keywords:** irritable bowel syndrome, probiotics, *Bifidobacterium lactis*, *Bacillus coagulans*, IBS-SSS

## Abstract

Probiotics offer a potential new therapeutic approach for irritable bowel syndrome (IBS), but current results are still controversial. The aim of this study was to assess the efficacy and safety of single-strain probiotic formulations in adult IBS patients and to compare the effects of *Bifidobacterium lactis* NORDBIOTIC™ BI040 (DSM 33812/34614) and *Bacillus coagulans* NORDBIOTIC™ BC300 (DSM 33836) in a prospective three-arm interventional randomized double-blind placebo-controlled clinical trial. The study included 123 IBS subjects diagnosed according to the Rome IV criteria. The primary outcomes were changes in symptom severity and symptom improvement as assessed using the IBS Severity Scoring System (IBS-SSS) after 4, 8, and 12 weeks of intervention and after 4 weeks of follow-up. Secondary outcomes included the assessment of individual IBS symptoms and the occurrence of adverse events. During the 12-week intervention, IBS-SSS scores significantly decreased (*p*-values < 0.001) in the study groups but differences between the interventional and placebo groups did not reach statistical significance. However, at the 16th week of follow-up, a significant improvement in the total IBS-SSS score in comparison to the placebo group (20.5%) was found in 43.8% and 52.9% of the *Bifidobacterium lactis* (*p* = 0.038, OR 3.0, [95% CI 1.1–8.6]) and the *Bacillus coagulans* (*p* = 0.005, OR 4.6 [95% CI 1.5–12.2]) groups, respectively. *Bifidobacterium lactis* had a beneficial effect on the intensity and frequency of pain, whereas *Bacillus coagulans* decreased the bowel dissatisfaction. Both strains increased the percentage of patients with normal stool consistency, but only *Bifidobacterium lactis* induced a decrease in the number of patients with constipation after 6 weeks of supplementation. Both probiotic strains were well tolerated, without differences in the occurrence of adverse events between groups. In conclusion, single-strain supplementation was safe and efficient in IBS patients but showed a different range of effects. *Bifidobacterium lactis* BI040 primarily reduced the frequency and intensity of pain, while *Bacillus coagulans* BC300 increased bowel satisfaction [ClinicalTrials.gov NCT05064930].

## 1. Introduction

Irritable bowel syndrome (IBS) is one of the most commonly diagnosed functional gastrointestinal disorders and is characterized by recurrent abdominal pain and discomfort associated with changes in bowel frequency and/or stool consistency in the absence of an organic disease [1]. The global prevalence of IBS ranges from <5% up to 20%, depending on the country or region and the criteria used [2,3]. IBS is classified on the basis of the stool pattern as diarrhea-predominant (IBS-D), constipation-predominant (IBS-C), mixed bowel habits (IBS-M), and un-classified (IBS-U). IBS mostly starts in early adulthood and is more common among women than men [1].

IBS pathogenesis is multifactorial with a spectrum of abnormalities, including mucosal inflammation, increased intestinal permeability, altered gut motility, visceral hypersensitivity, small-bowel bacterial overgrowth (SIBO), compromised gut microbiome compromised gut microbiome, and impaired neuroendocrinal communications [4,5,6,7,8]. It is currently supposed that gut microbiota dysbiosis may be a potential trigger for IBS, inducing most of the pathological conditions [7,8]. Indeed, analyses of stool samples of IBS patients in comparison with healthy controls showed decreased proportions of the genera *Bifidobacterium* and *Lactobacillus* and increased ratios of the phylum *Firmicutes*: *Bacteroidetes* in most studies [9,10,11]. Thus, the modulation of dysbiotic intestinal microbiota by biologically active biotics including probiotics, prebiotics, and, when combined, as synbiotics, is a promising treatment approach in IBS. Probiotics are defined as live microorganisms that, when administrated at the proper dose, have beneficial effects on host health [12]. Prebiotics (most often oligosaccharides such as fructo-oligosaccharides (FOS) or galacto-oligosaccharides) are substrates that are selectively utilized by host microorganisms and probiotic strains conferring a health benefit [13]. Clinical trials indicate that probiotic or synbiotic administration is beneficial for IBS patients, but the efficacy of such therapies depends on probiotic strain selection [14,15,16]. Our research team evaluated a multi-strain probiotic formulation and a multi-strain synbiotic (a combination of probiotic strains with short-chain prebiotic FOS) formulation in two randomized double-blind placebo-controlled (RDBPC) clinical trials in patients with IBS-D [17,18]. The results indicated a beneficial effect of both formulations on the clinical course of IBS, assessed using the international IBS symptom severity scale (IBS-SSS), but with each of the study formulations exhibiting effectiveness in different fields. The formulation composed of a mixture of *Bifidobacterium*, *Lactobacillus*, and *Streptococcus thermophilus* strains significantly reduced pain severity and improved the quality of patients’ life [17], whereas the synbiotic formulation effectively improved bloating and had a beneficial effect on the general condition of the intestines [18]. A systematic review and meta-analysis published in 2020 showed that single-strain formulations, particularly those containing *Bifidobacterium* or *Lactobacillus* strains, may be more effective in IBS patients [14]. Other reports demonstrated high efficacy of a new-generation probiotic—the lactic acid-producing *Bacillus coagulans* [19]. Therefore, the main objective of the current study is to assess the efficacy and safety of the single-strain probiotic formulation in patients with IBS. We would like to compare the effects of two different probiotic strains, i.e., *Bifidobacterium lactis* NORDBIOTIC™ BI040 and *Bacillus coagulans* NORDBIOTIC™ BC300 in a prospective three-arm interventional RDBPC clinical trial.

## 2. Materials and Methods

### 2.1. Study Design

This was a prospective interventional RDBPC trial. The participants consisted of patients attending gastroenterology outpatient clinics between September 2021 and July 2022. The study was conducted in accordance with the ethical principles set out in the Declaration of Helsinki Guideline on Good Clinical Practice. The trial was approved by the Bioethics Committee at the Regional Medical Chamber in Lublin and was given decision number 173/202l/KB/VIII. The participants were informed of the purposes and conditions of the study and signed the relevant informed consent form. The patients were also informed of the option to refuse and withdraw their consent at any time, without stating the reason or suffering any consequences, and without losing the right to receive further care at the outpatient clinics or at the Department of Gastroenterology, Medical University of Lublin. The study was registered at ClinicalTrials.gov and received the trial number NCT05064930.

**Figure 1:**
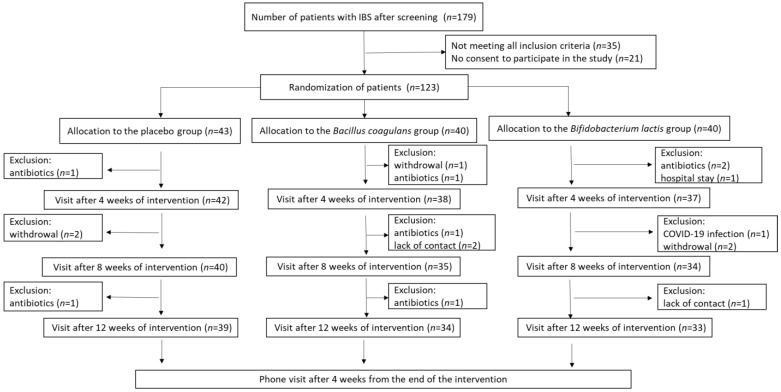
The flowchart of study protocol.

The study schedule included six visits: the screening visit, whose purpose was to qualify patients to be enrolled in the study; the baseline visit after up to 14 days following the screening visit, at which participants were randomized to the intervention groups; three follow-up visits after a 1-week run-in observation, held at weeks 4, 8, and 12 ± 3 days after starting the intervention; and the final follow-up visit after finishing the intervention at week 16. The last follow-up visit was a phone visit by the researchers; the other visits were at the clinics. The scheme of the study protocol is presented in Figure 1. After enrollment into the study, participants received a diary to fill out on a daily basis. Participants were also monitored weekly by interviewers by phone throughout the study.

#### 2.1.1. Patient Inclusion and Exclusion Criteria

The study included female and male patients aged 18–70 years who were diagnosed with IBS according to the Rome IV criteria, i.e., patients with recurrent abdominal pain on average at least one day a week in the last three months, associated with two or more of the following: (1) related to defecation (either increasing or improving pain), (2) associated with a change in stool frequency, (3) associated with a change in stool form (appearance) [20]. Stool consistency was assessed with the Bristol Stool Form (BSF) scale [21]. Patients with the following IBS types were included: IBS-D (more than 25% of BSF type 6 and 7 stools and less than 25% of type 1 and 2 stools); IBS-C (more than 25% of BSF type 1 and 2 stools, with less than 25% of type 6 and 7 stools); IBS-M (more than 25% of BSF type 6 and 7 stools and also more than 25% of type 1 and 2 stools). IBS severity was assessed by the use of the IBS-SSS score [22]. Patients with at least a moderate type of IBS (IBS-SSS score > 175 points) were enrolled into the study.

The exclusion criteria were subjects aged <18 and >70 years; unclassified IBS; gastrointestinal conditions other than IBS, such as inflammatory bowel diseases, celiac disease, gastroenteritis, gastric and duodenal ulcers; constipation; parasitic or bacterial intestinal infestation/infections; diagnosed lactose intolerance; hypersensitivity to food allergens; coexisting severe diseases such as malignancies, uncontrolled hypertension (blood pressure > 170/100 mmHg), uncontrolled diabetes mellitus (fasting blood glucose > 11 mmol/L), serious neurological disorders, psychosis, respiratory disorders (asthma, chronic obstructive pulmonary disease), hyper- or hypothyroidism, and hepatic, renal or cardiac dysfunctions; pregnancy or breastfeeding; being on gluten-free and low FODMAP (Fermentable Oligosaccharides, Disaccharides, Monosaccharides, And Polyols) diets; antibiotic therapy during the 1 month preceding the study; the current use of dietary supplements or drugs targeting the gut microbiota, such as probiotics, prebiotics, synbiotics, and short chain fatty acids, and refusal to undergo a 1-month washout period. Other exclusion criteria included the use of motility medications or dietary fiber supplements within 2 weeks before the study start, taking anti-coagulant drugs, plans to have surgery during the time of the study, a history of alcohol or drug abuse, and participation in another clinical trial during the 3 months prior the study entry. Patients who were treated with antibiotics during the study were also excluded. Patients were allowed to take spasmolytic drugs on an ad hoc basis and/or the low-dose antidepressants amitriptyline, nortriptyline or selective serotonin inhibitor at up to 25 mg per day.

Withdrawal criteria after enrollment into the study included compliance with probiotic or placebo supplementation of below 80%, non-attendance at the study visits, non-contact with the telephone interviewer, exclusion criteria found after enrollment, and any serious adverse event during the intervention period.

#### 2.1.2. Intervention

Patients received capsules with a probiotic formulation containing *Bifidobacterium lactis* NORDBIOTIC™ BI040 ((DSM 33812/DSM 34614) or *Bacillus coagulans* NORDBIOTIC™ BC300 (DSM 33836) or placebo. The placebo contained maltodextrin (starch hydrolysate), i.e., compounds present in probiotic products. All capsules were identical in size, color, texture, and taste and marked as product A, B, or C. The packaging of the products looked identical and had an inscription containing the title of the study, the approval number of the Bioethical Committee, and the expiry date. The probiotics or placebo were administered orally at 5 × 10^9^ *Bifidobacterium lactis* BI040 cells/day or 2 × 10^9^ *Bacillus coagulans* BC300 cells/day over a period of 12 weeks. Preparation of the probiotics and placebo, blinding of samples, and their delivery were performed by Nordic Biotic Sp z o.o., Poland. One batch of each product was produced and had a 2-year shelf life. The products were stored below 6 °C until they were delivered to researchers, where they were stored at room temperature. The products were refrigerated for no longer than 8 months and were issued to researchers every 2–3 months as needed.

#### 2.1.3. The Study Protocol

During screening visits, the patients underwent physical examination to establish the presence of clinical inclusion criteria. Out of 223 patients with IBS, 196 met the inclusion criteria for age and IBS severity (IBS-SSS score > 175), with 45 of them not meeting some of the other inclusion criteria and a further 31 not agreeing to participate in the study (Figure 1). Finally, 120 patients meeting all inclusion criteria signed the informed consent form and were enrolled into the trial. All participants were instructed on how to assess the symptoms about which they would be asked by telephone interviewers and were trained not to consume foods and dietary supplements containing probiotics/synbiotics or other active agents influencing the gut microbiota according to the exclusion criteria. During the first baseline visit, patients were allocated to groups A, B, or C according to a computer-generated randomization list with the use of the web page https://www.random.org/lists “URL (accessed on 20 August 2021)”. The randomization was blinded to both patients and researchers. At the baseline visit, the researchers informed participants not to take the product for the first week (7 days run-in observation) and to complete a diary daily (containing information about the number and type of stool and the severity of specific IBS symptoms); on the 7th day, a telephone interviewer informed the participant to start taking the product from the next day onwards. Patients were asked to take orally one capsule a day over a 12-week period. Patients reported to a researcher every 4 weeks in order to receive the probiotic preparation or placebo for the next 4 weeks and to be clinically assessed. The telephone interviewers called patients once a week and reminded patients to fill out diaries daily, and they also collected information on the IBS symptoms, drugs taken, and the occurrence of any adverse events. The telephone interviewers were recruited from among the research staff of the Department of Pathomorphology, the Children’s Memorial Health Institute in Warsaw.

#### 2.1.4. Outcome Definitions

The primary outcomes included an assessment of IBS severity with the use of the IBS-SSS score and any improvement of clinical symptoms. IBS-SSS is a 5-question survey about the severity of abdominal pain (IBS-SSS1), the number of days with abdominal pain over the last 10 days (IBS-SSS2), the severity of abdominal distension (IBS-SSS3), dissatisfaction with bowel habit (IBS-SSS4), and interference with the quality of life over the past 10 days (IBS-SSS5) [22]. The number of days with pain was multiplied by 10. Each of the five questions generate a maximum score of 100 points, and total scores ranged from 0–500, with higher scores indicating severe symptoms. A drop of at least 2 scoring categories (at least 50%) compared with the baseline was assumed to be associated with a clinically meaningful improvement. In the case of the number of days with pain, a decrease below at least 2 standard deviations was considered an improvement.

Secondary outcomes included changes in stool consistency evaluated using the BSF scale, the number of bowel movements per day, the severity of pain and flatulence, fecal urgency, the feeling of incomplete stool evacuation, and the effect of intervention on the occurrence of adverse events. The data were collected from patients’ diaries and telephone interviewers. IBS symptoms, except for the sensation of incomplete bowel movements, were assessed using a patient-defined 5-point Likert’s scale as was described in our previous paper [18]. Briefly, a score of 0 indicated no symptoms, and scores of 1–4 were based on the severity of symptoms; the higher the score, the more severe the symptoms. A feeling of incomplete bowel movement was assessed using a 2-point scale: 0 = no such feeling and 1 = there is an incomplete bowel movement. Adverse events and the taking of drugs were evaluated as either 0 = no or 1 = yes.

### 2.2. Statistics

The Stata Program version 16.0 was used for statistical analysis. Differences between the groups in nominal variables (e.g., sex, the number of patients with an improvement, adverse events, the absence or presence of specific symptoms) were evaluated with the use of Fisher’s exact test. In the case of continuous variables (e.g., age, physical development parameters, IBS-SSS scores, the duration of adverse events), ANOVA and Repeated Measures ANOVA (RM-ANOVA) were used if the normality of residuals assumption held. The normality of distribution of residuals was analyzed by the Shapiro–Wilk test. When the assumption of the normal distribution of residuals did not hold, the Kruskal–Wallis multiple comparison test and the Scheirer–Ray–Hare test were used. In the follow-up analysis of the pairwise comparisons, the Wilcoxon rank-sum test was applied. The threshold of significance for all analyses was set to α = 0.05. In the follow-up analysis of the pairwise comparisons, the *p*-values were adjusted with the use of the Bonferroni or Benjamini–Hochberg methods.

To reduce the dimension of the dataset from the patients’ diary (day) level, the data were transformed to weekly level observations. For all variables, except for a feeling of incomplete bowel movement and adverse events, the mean (average) value for each week was calculated. For a feeling of incomplete bowel movement, coded as a dummy variable taking values 0 or 1, the most common value was calculated for each week (mode). For side effects, two measures were calculated: (1) a variable for the presence of the adverse events at a week level (no matter how many days), and (2) the duration of side effects expressed as the number of days the side effect was observed during each week. In the second step, the scales used in diaries were transformed as follows: type of stools (1 = normal, 0 = constipation, or 2 = diarrhea), intensity of pain, flatulence, or fecal urgency (0 = no symptoms, 1 = moderate, or 2 = severe).

## 3. Results

### 3.1. Patients

A total of 123 patients were randomized to receive the probiotic preparation or the placebo (Figure 1). After the 12-week intervention period, 7 patients from the *Bifidobacterium lactis*-supplemented group dropped out of the study due to antibiotics use (n = 2), hospital stay (n = 1), COVID-19 infection (n = 1), lack of contact with the interviewer (n = 1), and resignation due to no improvement (n = 2), while 6 patients dropped out of the *Bacillus coagulans*-supplemented group due to antibiotics use (n = 3), lack of contact with the interviewer (n = 2), and resignation due to no improvement (n = 1). Four patients from the placebo group dropped out due to antibiotics use (n = 2) and resignation due to no improvement (n = 2).

A final total of 106 patients (86.7% of enrolled patients) finished the study. Characteristics of the patients are shown in Table 1. Females were predominant in all groups. The mean age of the patients ranged from 39.5 ± 13.8 years in the placebo group to 40.8 ± 13.2 in the *Bifidobacterium lactis* group and 39.0 ± 17.0 years in the *Bacillus coagulans* group. Most often, patients were diagnosed with IBS-M and IBS-D. The IBS-D type was most commonly observed in the *Bacillus coagulans* group (50.0%), while the IBS-M type was most common in the *Bifidobacterium lactis* and placebo groups, comprising 42.4% and 58.9% of the patients, respectively. Patients with severe IBS (with IBS-SSS score > 300 points) predominated in all groups, accounting for 76.9% in the placebo group, 69.7% in the *Bifidobacterium lactis* group, and 76.5% in the *Bacillus coagulans* group. Statistical analysis showed no statistically significant differences between the study groups in terms of patient sex, age, physical development, type of IBS, or IBS severity.

### 3.2. The Effect of Probiotic Supplementation on the IBS-SSS Score

The primary outcomes included changes in IBS symptom severity as evaluated with the use of the IBS-SSS score. The mean values of the total IBS-SSS score before the intervention were similar in all the study groups, being 349.7 ± 58.0, 344.4 ± 63.9 and 335.8 ± 65.1 in the placebo, *Bifidobacterium lactis* and *Bacillus coagulans* groups, respectively (Table 2). During the 12-week intervention, IBS-SSS scores steadily and significantly decreased (*p*-values < 0.001) in both the probiotic-supplemented groups (to 167.8 ± 85.8 and 184.2 ± 97.4 in the *Bifidobacterium lactis* and the *Bacillus coagulans* groups, respectively) and in the placebo group (180.5 ± 81.9). The observed strong placebo effect resulted in no statistically significant differences between the groups with the probiotic intervention and the control group, except for a statistically significant reduction in pain frequency after 4 weeks of intervention in the *Bifidobacterium lactis* group compared with the placebo group (*p*-value = 0.013). The average number of days with pain during last 10 days in the *Bifidobacterium lactis*-supplemented group decreased from 6.8 before the intervention to 3.7, while in the placebo group, it was 7.0 and 5.5, respectively. This decrease was maintained throughout the intervention period (12 weeks) and after its completion. At week 16 (4 weeks after the end of the intervention), the *Bifidobacterium lactis* group had a mean number of pain days of 2.6, which was significantly (*p*-value = 0.008) lower compared with the placebo group, where pain was reported for an average of 4.6 days. Interestingly, the number of days with pain in the *Bifidobacterium lactis* group at the 16-week follow-up observation was statistically significantly fewer even compared with the group supplemented with the *Bacillus coagulans* strain, in which the average number of pain-free days was 3.9 (*p*-value = 0.042). *Bifidobacterium lactis* BI040 supplementation also had a significant effect on pain intensity. In the 16th week of follow-up observation, the IBS-SSS2 pain intensity score was significantly lower in the *Bifidobacterium lactis* group compared with the placebo group (*p*-value = 0.026) and amounted to 25.0 ± 19.4 and 37.8 ± 19.8 points, respectively.

In the 16th week of the trial, i.e., 4 weeks after the end of the intervention, a decrease in the IBS-SSS scores compared with the baseline was observed in all study groups, but at the same time, an increase in the scoring, indicating a worsening of the clinical condition compared with week 12, i.e., the end of the intervention, was found. However, this increase in scores was primarily seen in the placebo group. Compared with the end of the intervention, the average total IBS-SSS score in the follow-up observation increased by 55.8 points in the placebo group, while it increased by 28.6 points in the *Bifidobacterium lactis* group and only by 8.6 points in the *Bacillus coagulans* group. The beneficial effect of probiotic supplementation after the end of intervention during the follow-up period was observed mainly in terms of the intensity and frequency of pain in the *Bifidobacterium lactis* group described above, and of the dissatisfaction with the bowel habit in the *Bacillus coagulans* group. Four weeks after the end of the intervention, bowel dissatisfaction was rated at an average of 47.0 points in the *Bacillus coagulans*-supplemented group, while in the placebo group, it was as high as 59.4 points (*p*-value = 0.044).

### 3.3. The Effect of Probiotic Intervention on Clinical Improvement

**Figure 2:**
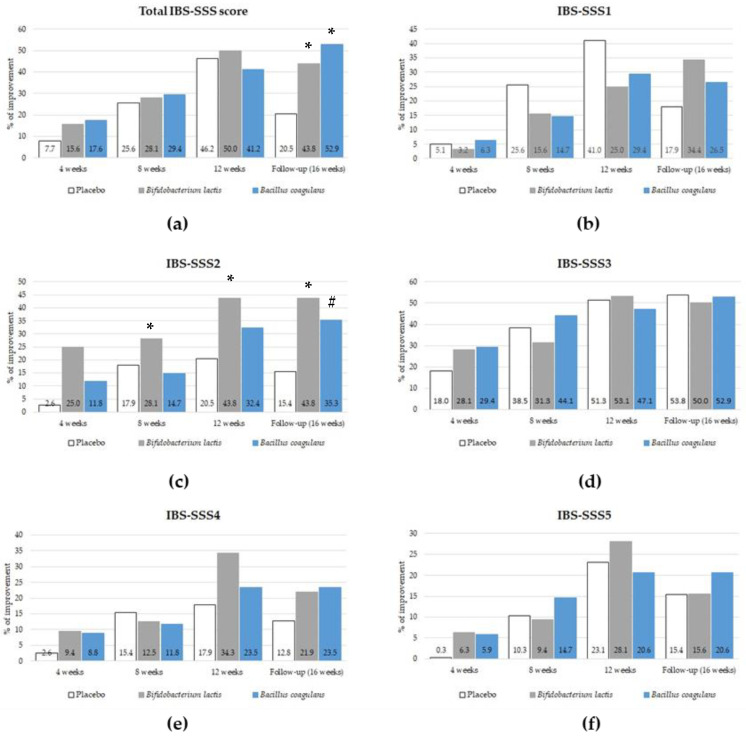
The effect of probiotic intervention on clinical improvement assessed by IBS-SSS scores (**a**–**f**). * indicates a statistically significant improvement (*p* < 0.05) between both the groups given probiotic intervention and the placebo group in the total IBS-SSS score (**a**), and between the *Bifidobacterium lactis* BI040 group and the placebo group in the IBS-SSS2 score assessing the frequency of pain (**c**); # *p*-value = 0.054 between the *Bacillus coagulans* BC300 group and the placebo group (**c**).

An improvement in clinical symptoms, defined as a drop in IBS-SSS scores by at least two scoring categories (at least by 50%) or a decrease the number of days without pain by 2 standard deviations compared with the baseline observation, was a primary endpoint of the current study. The effect of intervention on clinical improvement is presented in Figure 2, and detailed statistical analysis for significant results involving odds ratio (OR) and 95% confidence interval (CI) is shown in Table 3.

Statistically significant differences between study groups in an improvement was found for the total IBS-SSS score and the IBS-SSS2 score evaluating the frequency of pain (Table 3). A significant improvement in global clinical symptoms assessed with the total IBS-SSS score in comparison with the placebo group was observed in both probiotic-supplemented groups after the end of the intervention at the 16th week of the trial. At this follow-up visit, 52.9% and 43.8% of the *Bacillus coagulans*-supplemented (*p* = 0.005, OR 4.6 [95% CI 1.5–12.2]) and *Bifidobacterium lactis*-supplemented (*p* = 0.038, OR 3.0 [95% CI 1.1–8.6]) groups, respectively, reported clinical improvement compared with 20.5% in the placebo group. A significant improvement in the number of days with pain was observed after 4 weeks (*p* = 0.02, OR 12.7 [95% CI 1.5–107.7]) and 12 weeks (*p* = 0.038, OR 3.0 [95% CI 1.1–8.6]) of intervention only in the *Bifidobacterium lactis* group compared with the placebo group. This positive effect was maintained for a further 4 weeks after the end of the intervention (*p* = 0.01, OR 4.3 [95% CI 1.4–13.0]). Patients from the *Bacillus coagulans* group also reported an improvement in the frequency of pain during the follow-up visit at the 16th week of the trial, reaching a *p*-value of 0.054 (OR 3.0 [95% CI 0.98–9.2]) compared with the placebo group.

### 3.4. The Effect of Intervention on Secondary Outcomes

**Figure 3:**
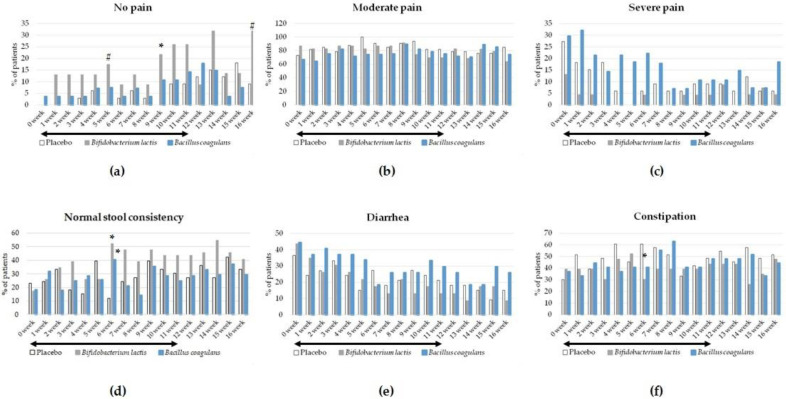
The effect of probiotic intervention on the intensity of pain, assessed using Likert’s scales (**a**–**c**), and the stool consistency, assessed with the Bristol Stool Formation scale (**d**–**f**). Data were reported daily in the patients’ diaries. * indicates statistically significant (*p* < 0.05) differences between the probiotic groups and the placebo group. # *p*-value < 0.08 and >0.05 between the probiotic groups and the placebo group. The black arrows show the duration of the intervention.

Secondary outcomes included daily data reported by the patients in their diaries. Patients assessed the frequency and consistency of their stool, the severity of pain and flatulence, fecal urgency, and the feeling of incomplete evacuation of stool. In addition, telephone interviewers checked the completion of the diaries once a week. The percentage of returned diaries was as follows: in the placebo group, 84.6%; in the group supplemented with *Bifidobacterium lactis*, 69.7%; and in the group supplemented with *Bacillus coagulans*, 82.4%. The results confirmed the beneficial effect of *Bifidobacterium lactis* on the pain feeling (Figure 3 and Table 4). At the baseline week (week 0), all patients but one in the *Bacillus coagulans* group reported the presence of pain. During the intervention, only in the *Bifidobacterium lactis* group did the percentage of patients with no pain increase, reaching a statistically significant difference in comparison with the placebo group at week 9 (*p* = 0.047, OR 19.92 [95% CI 1.04–380.67]). At the 5th and 16th weeks of follow-up observation, the *p*-value between the groups was 0.07 (OR 15.46 [95% CI 0.79–302.78]) and 0.051 (OR 4.37 [95% CI 0.99–19.26]), respectively (Table 4). In contrast, such a beneficial effect on the pain feeling was not observed in the group supplemented with *Bacillus coagulans*. There were no significant differences in the intensity of pain (severe or moderate) between the study groups.

Probiotic intervention resulted also in a significant improvement of stool consistency as assessed with the use of the BSF scale, which was observed at the 6th week of intervention (Figure 3, Table 4). The percentage of patients who reported normal stool consistency significantly increased in both supplemented groups compared with the placebo group at week 6 and was 52.2% in the *Bifidobacterium lactis* group (*p* = 0.002, OR 7.91 [95% CI 2.10–29.83]), 40.7% in the *Bacillus coagulans* group (*p* = 0.015, OR 4.98 [95% CI 1.36–18.23]), and only 12.1% in the placebo group. In addition, probiotic intervention with *Bifidobacterium lactis* significantly decreased the number of patients with constipation at the 6th week of intervention compared with the placebo (*p* = 0.01, OR 0.24 [95% CI 0.08–0.73]). Probiotic intervention did not affect the occurrence of diarrhea at that period of intervention. Other secondary outcomes (the number of stools per day, the intensity of flatulence, fecal urgency, and the feeling of incomplete evacuation of stool) were not affected by the probiotic interventions.

### 3.5. Safety and Adverse Events

**Figure 4:**
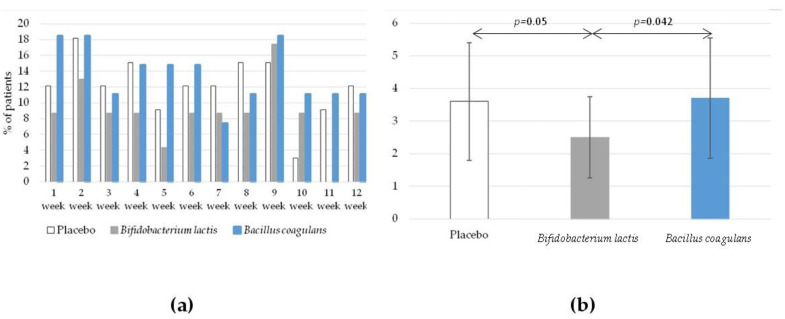
The percentage of patients reporting adverse events (**a**) and the mean number of days with adverse events (**b**) during an intervention period.

Adverse events were reported in the patient’s diaries, and the data were collected by the telephone interviewers. The data obtained from both sources were converged and showed that both the *Bifidobacterium lactis* BI040 and *Bacillus coagulans* BC300 probiotics were safe and well tolerated. There were no statistically significant differences in the percentage of patients reporting adverse events between the study groups (Figure 4). Interestingly, patients supplemented with *Bifidobacterium lactis* reported a shorter duration of adverse events in comparison with the placebo group (*p* = 0.05) as well as with the *Bacillus coagulans* group (*p* = 0.042). The mean number of days with adverse events in the *Bifidobacterium lactis* group was 2.5 ± 1.9, whereas in the placebo group, it was 3.7 ± 2.0, and in the *Bacillus coagulans* group, it was 3.8 ± 2.4 (Figure 4).

## 4. Discussion

Probiotics are potentially a promising approach in the treatment of functional bowel disorders, including IBS; however, discussions about their efficacy, dose, and composition are ongoing. The efficacy and safety of the use of probiotic products for IBS are supported by an increasing number of RDBPC studies. However, meta-analyses of these clinical trials do not give a clear answer as to which probiotics can be recommended in the treatment of IBS [14,15,16,19]. A meta-analysis of 37 RDBPC trials with 4403 patients published by Ford et al. in 2018 presented evidence for the use of combinations of probiotics as a group for improving global IBS symptoms and abdominal pain [15]. They also presented a trend toward a beneficial effect of *Bifidobacterium strains*. In terms of single-strain probiotics, *Lactobacillus plantarum* DSM 9843, *Escherichia coli* DSM1752, and *Streptococcus faecium* appeared to be beneficial, but the authors emphasized that the latter two were only used in one RDBPC trial. Xie et al. tried to evaluate the most effective combinations and components among different probiotics through a network meta-analysis of 65 clinical trials [23]. Standard network meta-analyses showed that *Lactobacillus* and *Bifidobacterium strains* were the most effective for the relief of global IBS symptoms and an improvement in abdominal pain. Component network meta-analyses showed that *Bacillus* and *Lactobacillus* were among the most effective components. A recently published network meta-analysis by Zhang et al. of 43 RDPC trials with 5531 IBS patients comparing different species showed that *Bacillus coagulans* exhibited the highest probability of being the optimal probiotic species for improving the IBS symptom relief rate as well as global symptoms, abdominal pain, bloating, and straining scores [19]. In addition, *Bacillus coagulans* had also significant efficacy compared with different types of probiotic combinations. Although different species and combinations of probiotics used in clinical trials were evaluated in the network meta-analyses, there are exceptions where studies compared the effectiveness of different species/strains during the one clinical trial [24]. That is why we decided to compare the effectiveness of two single-strain probiotic preparations in a three-arm interventional study involving adult IBS patients. We analyzed the effect of *Bacillus coagulans* NORDBIOTIC™ BC300, which was presented as the species with the highest efficacy in IBS patients [19], and *Bifidobacterium lactis* NORDBIOTIC™ BI040, the species which, when used in probiotic combinations in our earlier study in IBS-D patients, was demonstrated to improve the intensity of abdominal pain and the quality of the patients’ lives [17]. The current study has shown that both the *Bifidobacterium lactis* BI040 and *Bacillus coagulans* BC300 strains beneficially affect clinical symptoms in IBS patients but in a different range. Both strains improved the total IBS-SSS score, but *Bifidobacterium lactis* BI040 significantly reduced the frequency and intensity of pain, whereas *Bacillus coagulans* BC300 mainly ameliorated satisfaction from the bowel. Both strains beneficially affected the stool consistency, increasing the percentage of patients with normal stools as assessed with the BSF scale, but only *Bifidobacterium lactis* BI040 supplementation significantly decreased the number of patients with constipation in comparison with the placebo.

The obtained results, especially regarding the effect of *Bacillus coagulans* BC300 supplementation on IBS symptoms, are not fully consistent with the results of other researchers. Madempudi et al. showed that *Bacillus coagulans* Unique IS2 was efficacious in reducing abdominal pain and other IBS associated symptoms such as bloating, incomplete evacuation, urgency, bowel habit satisfaction, and stool consistency in adult IBS patients diagnosed according to the Rome III criteria (in our study, IBS patients were diagnosed according to the more restrictive Rome IV criteria) [25]. Gupta and Moity showed that *Bacillus coagulans* LBS improved most IBS symptoms, including the abdominal pain in an intervention group compared with the placebo group [26]. Similar results were obtained when IBS-D patients were supplemented with *Bacillus coagulans* MTCC 5856 [27]. Contrary to these results, our research showed that supplementation of IBS patients with *Bacillus coagulans* BC300 resulted in a statistically significant improvement in the global IBS symptoms as assessed by the total IBS-SSS score, but the improvement in the frequency of pain (the IBS-SSS2 score) showed only a statistically significant trend in comparison with the placebo group. The observed differences in results may be due to different ways of assessing IBS symptoms. In our study, we also used the IBS-SSS score in relation to individual symptoms. It should be emphasized that in our analysis, we assumed that an improvement occurs when there is a change in the IBS-SSS score by at least two point categories, i.e., by at least 50% compared with the baseline score. Gupta and Moity assessed the frequency of IBS symptoms with the use of the Digestive Symptom Frequency Questionnaire on a 5-point Likert scale (0 = never, 1 = ≤1 episode/week; 2 = ≤3 episodes/week; 3 = ≥3 episodes/week; 4 = daily episodes) [26], whereas Madenpudi et al. measured pain intensity on an 11-point numerical rating scale [25], and Maajed et al. used a 10 cm visual analog scale [27]. It should be emphasized that when stool consistency was assessed using the same BSF scale, the results did not differ. Similarly to other authors, we observed a beneficial effect of *Bacillus coagulans* BC300 supplementation on the normalization of stool consistency compared with the placebo group.

The current trial shows that supplementation with *Bifidobacterium lactis* BI040 results in a significant improvement in the clinical condition of IBS patients compared with the placebo group, mainly due to a reduction in the frequency and intensity of pain and the normalization of stool consistency. As in our study, Martoni et al. showed that *Bifidobacterium lactis* UABla-12 significantly reduced abdominal pain severity in IBS patients comparing with the placebo [24]. It is interesting that our earlier study of the effectiveness of combinations of probiotics containing *Bifidobacterium lactis* Bl040 in IBS-D patients showed that the administration of this multi-strain probiotic, like the single strain in the current study, has a beneficial effect on pain perception [17]. Such an effect was not observed in our other study conducted according to the same protocol, in which a different combination of probiotic strains, without *Bifidobacterium lactis* Bl040, was used [18]. In this study, we observed a beneficial effect mainly on flatulence.

We showed that *Bifidobacterium lactis* Bl040, like *Bacillus coagulans* BC300, significantly increased the percentage of IBS patients with normal stool consistency when compared with the placebo, but only supplementation with the *Bifidobacterium lactis* BI040 strain decreased the number of patients with constipation, suggesting that only *Bifidobacterium lactis* BI040 has a beneficial effect on constipation in IBS patients. This result is in line with a recently published meta-analysis evaluating the efficacy of probiotics in patients with chronic constipation [28]. The authors demonstrated that probiotics increased stool frequency, with *Bifidobacterium lactis* having a significant effect, but not combinations of probiotics or single strains, such as *Bacillus coagulans* Unique IS2 and *Lactobacillus casei* Shirota.

In the current study, we have observed a strong placebo effect. Patients in the placebo group reported significant improvement of IBS symptoms as assessed by the IBS score. This effect can be explained primarily by the influence of the weekly telephone contacts, during which patients were asked in great detail about their symptoms and the effects of treatment. This is also confirmed by the observation that the beneficial effect of treatment only in the placebo group changed trend after the end of therapy. It was only after a month of follow-up observation that we observed most of the statistically significant differences between the intervention groups and the placebo group. On the other hand, telephone contact made it possible to control the regularity of taking the product, the weekly assessment of product tolerance, and side effects. However, we cannot rule out that the maltodextrin used as a placebo in the present study could have had a beneficial effect on patients in the placebo group. Almutairi et al., in a meta-analysis of randomized RDBPC trials, showed that orally consumed maltodextrin often (in 61.8% of analyzed trials) induced alterations in the gut microbiome, including changes in the *Firmicutes* and/or *Bacteroidetes* phyla and *Lactobacillus* and/or *Bifidobacterium* species [29]. As we assume that the intestinal microbiota plays a key role in the pathogenesis of IBS, the strong placebo effect observed in our study may be due to the direct positive effect of maltodextrin.

### Strengths and Limitations of the Study

The strength of our study is that it was an RDBPC trial conducted by a team of researchers with extensive experience in this field (the researchers had conducted two other RDBPC trials evaluating the effectiveness of multi-strain probiotics in patients diagnosed with IBS-D, the results of which have been published) [17,18]. The efficiency of probiotics was assessed using IBS-SSS scores, the BSF scale, and Likert’s scales, and the safety of the probiotics was monitored during the 12-week intervention. The current study is unique in that it is one of the few studies that simultaneously evaluates the effect of two different single-strain probiotics in a three-arm intervention study, which allows for comparing effects in the same population of subjects.

Despite the numerous strengths of the study, the authors are aware of the presence of certain limitations, which include the lack of assessment of the intervention on the composition of the intestinal microbiome, which could explain the impact of both probiotic strains and maltodextrin on the gut microbiota of IBS patients. One limitation is undoubtedly the lack of assessment of the impact of the intervention on the degree of stress and anxiety associated with treatment, which could explain the strong placebo effect.

## 5. Conclusions

The current three-arm RDBPC interventional trial shows that both *Bifidobacterium lactis* NORDBIOTIC™ Bl040 and *Bacillus coagulans* NORDBIOTIC™ BC300 are safe and well tolerated by IBS patients and effectively improve global IBS symptoms as assessed by the IBS-SSS score, but that they have a different range of effects. Supplementation with *Bifidobacterium lactis* BI040 significantly reduced the frequency and intensity of pain compared with the placebo group, while *Bacillus coagulans* BC300 ameliorated the satisfaction from the bowel. Both strains had a positive effect on stool consistency. The results show species/strain-specific effectiveness in IBS patients and underline the role of proper selection of probiotics for the best approach according to patients’ specific needs.

## Figures and Tables

**Table 1:** Patients’ characteristics.

	Control Group (n = 39) N (%) or Mean ± SD	*Bifidobacterium lactis* NORDBIOTIC™ BI040 Group (n = 33) n (%) or Mean ± SD	*Bacillus coagulans* NORDBIOTIC™ BC300 Group (n = 34) n (%) or Mean ± SD
Gender			
Female	33 (84.6%)	21 (63.6%)	23 (67.4%)
Male	6 (15.4%)	11 (33.3%)	11 (32.6%)
Age in years	39.5 ± 13.8	40.8 ± 13.2	39.0 ± 17.0
Height (m)	1.68 ± 0.08	1.70 ± 0.13	1.69 ± 0.1
Body weight (kg)	67.0 ± 15.6	74.0 ± 16.9	70.8 ± 13.9
BMI	23.5 ± 4.2	25.7 ± 5.5	24.6 ± 4.5
IBS type			
IBS-D	12 (30.8%)	12 (36.4%)	17 (50.0%)
IBS-C	4 (10.3%)	7 (21.2%)	4 (11.7%)
IBS-M	23 (58.9%)	14 (42.4%)	12 (35.3%)
IBS severity *			
Moderate	9 (23.1%)	10 (30.3%)	8 (23.5%)
Severe	30 (76.9%)	23 (69.7%)	26 (76.5%)
Total IBS-SSS score	349.7 ± 58.0	344.4 ± 63.9	335.8 ± 65.1

* IBS severity was assessed based on the IBS-SSS scale. There were no significant statistical differences between patients receiving probiotic preparations and the placebo control group. BMI = Body Mass Index. Severe IBS is when the IBS-SSS score is >300, moderate IBS is when IBS-SSS score is >175 and ≤300. SD = standard deviation.

**Table 2:** The effect of probiotic intervention on the severity of IBS symptoms assessed with the use of IBS-SSS scores.

Groups	Baseline	4 Weeks of Intervention		8 Weeks of Intervention		12 Weeks of Intervention		Follow-up after the End of Intervention (16 Weeks)	
	Mean ± SD	Mean ± SD	*p*-Value between Groups	Mean ± SD	*p* Value between Groups	Mean ± SD	*p*-Value between Groups	Mean ± SD	*p*-Value between Groups
IBS-SSS total score									
Placebo (P)	349.7 ± 58.0	277.6 ± 72.2	Bl vs. P: NS	224.7 ± 77.7	BI vs. P: NS	180.5 ± 81.9	BI vs. P: NS	236.3 ± 97.4	BI vs. P: NS
*Bifidobacterium lactis* BI040 (BI)	344.4 ± 63.9	241.1 ± 69.2	BC vs. P: NS	214.5 ± 80.2	BC vs. P: NS	167.8 ± 85.8	BC vs. P: NS	196.4 ± 89.6	BC vs. P: NS
*Bacillus coagulans* BC300 (BC)	335.8 ± 65.1	248.1 ± 89.8	Bl vs. BC: NS	212.7 ± 90.2	BI vs. BC: NS	184.2 ± 97.4	BI vs. BC: NS	192.8 ± 88.1	BI vs. BC: NS
IBS-SSS1 score (the intensity of pain)									
Placebo (P)	55.1 ± 19.2	39.1 ± 18.0	BI vs. P: NS	31.4 ± 17.9	BI vs. P: NS	24.4 ± 16.7	BI vs. P: NS	37.8 ± 19.8	* BI vs. P: 0.026
*Bifidobacterium lactis* BI040 (BI)	47.7 ± 17.0	29.6 ± 13.2	BC vs. P: NS	26.5 ± 14.0	BC vs. P: NS	22.7 ± 17.0	BC vs. P: NS	25.0 ± 19.4	BC vs. P: NS
*Bacillus coagulans* BC300 (BC)	49.3 ± 22.6	35.3 ± 21.4	BI vs. BC: NS	32.4 ± 20.0	BI vs. BC: NS	25.7 ± 19.9	BI vs. BC: NS	29.6 ± 22.9	BI vs. BC: NS
IBS-SSS2 score (the frequency of pain)									
Placebo (P)	70.0 ± 25.1	55.4 ± 24.9	* BI vs. P: 0.013	44.1 ± 32.3	BI vs. P: NS	33.3 ± 28.9	BI vs. P: NS	46.1 ± 30.1	* BI vs. P: 0.008
*Bifidobacterium lactis* BI040 (BI)	68.5 ± 27.3	37.6 ± 27.9	BC vs. P: NS	33.9 ± 26.0	BC vs. P: NS	23.9 ± 24.6	BC vs. P: NS	26.1 ± 18.9	BC vs. P: NS
*Bacillus coagulans* BC300 (BC)	66.8 ± 26.7	49.1 ± 29.7	BI vs. BC: NS	42.1 ± 26.7	BI vs. BC: NS	33.5 ± 26.8	BI vs. BC: NS	39.1 ± 24.3	* BI vs. BC: 0.042
IBS-SSS3 score (the severity of flatulance)									
Placebo	69.9 ± 28.2	50.0 ± 27.5	BI vs. P: NS	41.7 ± 19.3	BI vs. P: NS	31.4 ± 19.6	BI vs. P: NS	37.8 ± 22.8	BI vs. P: NS
*Bifidobacterium lactis* BI040 (BI)	70.4 ± 26.1	47.7 ± 24.5	BC vs. P: NS	43.9 ± 25.0	BC vs. P: NS	31.1 ± 17.7	BC vs. P: NS	38.7 ± 24.0	BC vs. P: NS
*Bacillus coagulans* BC300 (BC)	67.6 ± 22.6	47.1 ± 26.7	BI vs. BC: NS	35.3 ± 23.1	BI vs. BC: NS	31.6 ± 24.8	BI vs. BC: NS	31.1 ± 17.7	BI vs. BC: NS
IBS-SSS4 score (dissatisfaction with bowel habit)									
Placebo (P)	79.9 ± 16.9	68.7 ± 19.5	BI vs. P: NS	52.5 ± 18.2	BI vs. P: NS	46.6 ± 22.4	BI vs. P: NS	59.4 ± 24.4	BI vs. P: NS
*Bifidobacterium lactis* BI040 (BI)	81.4 ± 17.2	62.1 ± 18.2	BC vs. P: NS	56.1 ± 19.5	BC vs. P: NS	46.0 ± 20.1	BC vs. P: NS	51.2 ± 26.8	* BC vs. P: 0.044
*Bacillus coagulans* BC300 (BC)	80.0 ± 18.9	60.3 ± 20.9	BI vs. BC: NS	54.4 ± 23.0	BI vs. BC: NS	47.6 ± 26.1	BI vs. BC: NS	47.0 ± 18.5	BI vs. P: NS
IBS-SSS5 score (quality of life)									
Placebo (P)	74.7 ± 15.0	64.4 ± 15.2	BI vs. P: NS	55.0 ± 20.6	BI vs. P: NS	44.9 ± 24.6	BI vs. P: NS	55.1 ± 26.8	BI vs. P: NS
*Bifidobacterium lactis* BI040 (BI)	76.3 ± 17.9	64.1 ± 18.6	BC vs. P: NS	54.1 ± 24.6	BC vs. P: NS	44.0 ± 27.0	BC vs. P: NS	55.4 ± 19.9	BC vs. P: NS
*Bacillus coagulans* BC300 (BC)	72.1 ± 17.6	56.4 ± 23.9	BI vs. BC: NS	48.6 ± 27.3	BI vs. BC: NS	45.7 ± 28.3	BI vs. BC: NS	46.1 ± 24.8	BI vs. BC: NS

IBS symptoms were evaluated with the use of the IBS-SSS scores at baseline, 4th, 8th, and 12th week of the intervention, and at 4 weeks after the end of the intervention (16th week of the study). The table shows mean values of the IBS-SSS scores ± standard deviations (SD). A score reduction corresponded to symptom amelioration. * Statistically significant *p*-values (*p* < 0.05). NS = not significant.

**Table 3:** The effect of probiotic intervention on the improvement of IBS symptoms assessed by the total IBS-SSS score and IBS-SSS2 score.

Groups	4 Weeks of Intervention		8 Weeks of Intervention		12 Weeks of Intervention		Follow-Up (16 Weeks)	
	*p*-Value	OR (95% CI)	*p*-Value	OR (95% CI)	*p*-Value	OR (95% CI)	*p*-Value	OR (95% CI)
An improvement in clinical symptoms assessed by total IBS-SSS score								
*Bifidobacterium lactis* BI040 vs. placebo	NS	2.4 (0.5–10.9)	NS	1.1 (0.4–3.2)	NS	1.2 (04–3.0)	* 0.038	3.0 (1.1–8.6)
*Bacillus coagulans* BC300 vs. placebo	NS	2.7 (0.6–12.1)	NS	1.2 (0.4–3.4)	NS	0.8 (0.3–2.1)	* 0.005	4.6 (1.5–12.2)
An improvement in pain frequency assessed by IBS-SSS2 score								
*Bifidobacterium lactis* BI040 vs. placebo	* 0.020	12.7 (1.5–107.7)	NS	1.8 (0.6–5.5)	* 0.038	3.0 (1.1–8.6)	* 0.010	4.3 (1.4–13.0)
*Bacillus coagulans* BC300 vs. placebo	NS	5.1 (05–47.7)	NS	0.8 (0.2–2.7)	NS	1.8 (0.6–5.3)	0.054	3.0 (0.98–9.2)

The table shows the exact statistical analysis including p-value, odds ratio (OR) and 95% confidence interval (CI) of the clinical improvement (a decrease in IBS-SSS scores by at least 50% compared with baseline) between the groups with probiotic intervention and the placebo group. Only the analysis of parameters that showed statistically significant differences, i.e., total IBS-SSS score and the IBS-SSS2 score evaluating the frequency of pain. Clinical assessment using the IBS-SSS1, IBS-SSS3, IBS-SSS4, and IBS-SSS5 scores did not show any statistical differences between the study groups. * Statistically significant *p*-values (*p* < 0.05). NS = not significant.

**Table 4:** The effect of probiotic intervention on secondary outcomes, presenting statistical differences in comparison with the placebo.

Symptoms	*Bifidobacterium lactis* NORDBIOTIC™ BI040 Group (BI) n = 23	*Bacillus coagulans* NORDBIOTIC™ BC300 Group (BC) n = 27	Placebo Group (P) n = 33	OR [95% CI] *p*-Value	
				BI vs. P	BC vs. P
The pain intensity at 5th week of intervention					
No pain	4 (17.4%)	2 (7.4%)	0	15.46 [0.79–302.78] *p* = 0.07	NS
Severe	0	5 (18.5%)	0	NS	NS
Moderate	19 (82.6%)	20 (74.1%)	33 (100%)	NS	NS
The pain intensity at 9th week of intervention					
No pain	5 (21.7%)	3 (11.1%)	0	19.92 [1.04–380.67] *p* = 0.047	NS
Severe	1 (4.3%)	2 (7.4%)	2 (6.1%)	NS	NS
Moderate	17 (74.0%)	22 (81.5%)	31 (93.9%)	NS	NS
The pain intensity at 16th week of the trial (4 weeks after the end of the intervention)					
No pain	7 (30.4%)	2 (7.4%)	3 (9.1%)	4.37 [0.99–19.26] *p* = 0.051	NS
Severe	1 (4.3%)	5 (18.5%)	2 (6.1%)	NS	NS
Moderate	15 (65.2%)	20 (74.1%)	28 (84.8%)	NS	NS
Type of stool at 6th week of intervention					
Normal	12 (52.2%)	11 (40.7%)	4 (12.1%)	7.91 [2.10–29.83] *p* = 0.002	4.98 [1.36–18.23] *p* = 0.015
Constipation	7 (30.4%)	11 (40.7%)	20 (60.6%)	0.24 [0.08–0.73] *p* = 0.01	NS
Diarrhea	4 (17.4%)	5 (18.5%)	9 (27.3%)	NS	NS

The table shows the number and the percentage (in brackets) of patients reporting symptoms (the intensity of pain and the stool consistency) that showed statistical significant differences between groups supplemented with probiotics and the placebo group, the odds ratio (OR) and the 95% confidence interval [CI]. Data were reported daily in patients’ diaries. The intensity of pain was assessed using Likert’s scales and the stool consistency with the Bristol Stool Formation scale. Other symptoms assessed as secondary outcomes (the intensity of flatulence, fecal urgency, and the feeling of incomplete evacuation of stool) did not show statistical significantly differences between the study groups during the 16 weeks of observations and are not presented. NS = not significant.

## Data Availability

The description of the protocol study is available at https://clinicaltrials.gov “URL (accessed on 21 July 2023)” under the number NCT05064930. The data presented in this study are available on request from the corresponding author. The data are not publicly available due to privacy protection.
